# Systematically Investigating the Pharmacological Mechanism of *Momordica grosvenori* in the Treatment of Spinal Cord Injury by Network Pharmacology and Experimental Verification

**DOI:** 10.1155/2023/1638966

**Published:** 2023-01-25

**Authors:** Jiling Wang, Zihong Yang, Jie Jiang, Yang Xv, Xiuwei Tan, Ruyu Chen, Fengxin Li, Changqiu Li, Yiji Su

**Affiliations:** ^1^The First Clinical Medical College of Guangxi Medical University, Nanning, China; ^2^The Second Affiliated Hospital of Guangxi Medical University, Nanning, China; ^3^The First Affiliated Hospital of Guangxi Medical University, Nanning, China; ^4^Guangxi Research Center for Regenerative Medicine, Nanning, China

## Abstract

**Objective:**

This study aimed to explore the molecular mechanism of *Momordica grosvenori* (MG) in spinal cord injury (SCI) by network pharmacology analysis.

**Methods:**

We searched for potential active MG compounds using the TCMSP database and the BATMAN-TCM platform. The Swiss target prediction database was used to find MG-related targets and the targets of SCI from the CTD, GeneCards, and DrugBank databases. Following that, a protein-protein interaction (PPI) study was carried out. Cytoscape software was used to calculate the hub gene, and R software was used to evaluate the Gene Ontology (GO) and KEGG enrichment pathways. Finally, molecular docking between the hub protein and important compounds was performed. We verified STAT3, MAPK1, HSP90AA1, PIK3R1, PIK3CA, and RXRA potential targets by quantitative PCR.

**Results:**

We obtained 293 MG-anti-SCI targets with potential therapeutic utility by intersecting 346 MG-related targets and 7214 SCI-related targets. The top 10 identified genes, ranking in descending order of value, were SRC, STAT3, MAPK1, HSP90AA1, PIK3R1, PIK3CA, RXRA, AKT1, CREBBP, and JAK2. Through enrichment analysis and literature search, 10 signaling pathways were screened out. The molecular docking of important drugs and hub targets revealed that some had a higher binding affinity. The results of quantitative PCR indicated that MAPK1, RXRA, and STAT3 were expressed differently in in vitro experiments.

**Conclusion:**

In conclusion, the current work indicated that MG might play an anti-SCI role via multicomponent, multitarget, and multichannel interaction, which presents a novel idea for further research into the precise mechanism of MG-anti-SCI interaction.

## 1. Introduction

Spinal cord injury (SCI) is defined as structural damage and loss of whole or partial functional impairment of the spinal cord produced by various factors, often resulting in quadriplegia, paraplegia, or defecation dysfunction. The incidence of SCI on a global scale ranges from 10.4 to 83 cases per million people per year. SCI can affect patients' quality of life and consume plenty of healthcare resources [1, 2]. Complications such as lung infection, urinary tract infection, and pulmonary embolism are common after traumatic SCI and might result in death if the situation is severe [3, 4]. SCI has significantly harmed society and individuals, becoming a major worldwide health and medical issue [2, 5].

Clinical medical research has traditionally focused on the best approaches to treat spinal cord injuries. Within eight hours of the injury, methylprednisolone (MPS) can be utilized in some contraindication-free SCI cases, and patients should be advised of potential consequences [3]. Studies, however, indicate that MPS may increase gastrointestinal bleeding and that it provides no appreciable long-term benefits for those with acute traumatic SCI [6]. High-dose MPS treatment may increase the risk of adverse events in acute SCI patients but may not improve neurological recovery. Therefore, we advise against consistently using high-dose MPS early in acute SCI [7]. Riluzole can substantially improve the motor score, locomotor function, and neuropathic pain in the preclinical model of SCI [8]; however, insufficient data suggest riluzole's therapeutic efficacy for SCI. The effect of riluzole on traumatic and nontraumatic SCI is only verified in preclinical models [8]. Single-cell treatment for SCI has few advantages [9]. Numerous promising studies are being conducted to alleviate the severe effects of SCI. Positive clinical results have not yet been validated, and most trials are still in adolescence [10]. We need further study, as was already stated, to investigate and verify potential treatments.

Traditional Chinese medicine (TCM) has attracted the attention of more and more researchers in recent years as a potential treatment for SCI [11]. Additionally, the current study suggests that TCM is a successful treatment for SCI [11]. Our objective has always been to create efficient, affordable, and secure drugs. Long utilized as a food and medicine equivalent by the populace, MG is a perennial herbaceous vine of the Cucurbitaceae family that is exclusive to China. It is also very safe and reasonably priced. It is mostly grown in the Chinese province of Guangxi, in the counties of Yongfu, Lingui, and Longsheng. In Guangxi's traditional medicine, MG is homologous in medicine and food. It acts as an antioxidant [12], a hypoglycemic [13], an antitussive [14], a sputum reducer [15], an anti-inflammatory [16], an antimicrobial [17], etc. Kaempferol is one of the active ingredients of MG, which has neuroprotective [18], cardioprotective [19], anti-inflammatory [20], antioxidant [21], and anticancer effects [22]. Kaempferol can promote the recovery of motor function in SCI rats [23], indicating that MG may have great potential to repair SCI.

Nevertheless, MG's primary components and pharmacological effects have not been thoroughly identified, limiting future research and application. As a result, a thorough examination of MG's primary pharmacological components and implications is necessary. System analytic methodologies, such as network pharmacology, are promising for developing fresh approaches to elucidating the drug-gene-disease relationship [24]. The network pharmacology approach of TCM presents a novel study methodology [25]. This strategy will promote the use of evidence-based medicine in TCM, helping to prove MG's therapeutic value and improving the current drug discovery process [21].

**Figure 1:**
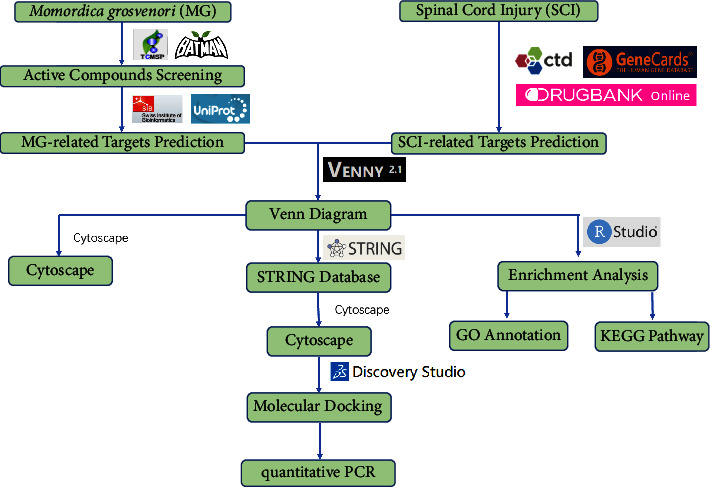
The overall flowchart of this study.

The study aimed to develop an analytical approach for validating the effects of MG on SCI using network pharmacology, molecular docking, and experiments.  Figure 1 depicts the workflow used in this study. The study plan intends to achieve the following objectives: (1) Discovering bioactive components in MG; (2) predicting the related targets of MG and SCI; (3) identifying SCI and MG-related biological processes, potential therapeutic targets, molecular mechanisms, and signal pathways using comprehensive network analysis; (4) and validating the above-analyzed data through molecular docking. Therefore, in the investigation of this study, we used monomer components of MG for experimental verification in consideration of better efficacy and evaluation methodologies.

## 2. Materials and Methods

### 2.1. Chemical Constructions of MG

All of the compounds in MG were searched by using databases and scientific studies, including Traditional Chinese Medicine Systems Pharmacology (TCMSP, https://tcmsp-e.com/) [14], a bioinformatics analysis tool for molecular mechanism of TCM (BATMAN-TCM, https://bionet.ncpsb.org.cn/batman-tcm/) [26]. In total, 182 compounds were obtained for further analysis.

### 2.2. Potential Active Compound Screening

As TCM, it is very important to screen drugs with appropriate ADME characteristics [27]. The percentage of oral medications that are absorbed by the digestive tract and reach the systemic circulation blood in an oral dose is referred to as oral bioavailability (OB) [28]. The term “drug-like properties value” (DL) refers to the similarity between compounds and known drugs [29]. The probability that a compound will become a drug increases with its DL. The pharmacological activity must occur under certain parameters, including appropriate OB and DL values. According to the TCMSP database, we must utilize OB ≥ 30% and DL ≥ 0.18 as ADME guideline ranges. As a result, we used OB ≥ 30% and DL ≥ 0.18 as screening criteria to identify potential compounds for further investigation.

### 2.3. Target Identification

The predicted targets of active compounds screened from MG were obtained from the SwissTargetPrediction database (https://www.SwissTargetPrediction.ch/) [30]. The final therapeutic target was then obtained by converting the target into UniProt ID and removing duplicate values.

### 2.4. Screening of SCI Targets

We obtained potential targets of SCI from the GeneCards database (https://www.genecards.org/) [31], the Comparative Toxicogenomics database (CTD, https://ctdbase.org/) [32], and the DrugBank database (https://go.drugbank.com/) [33].

### 2.5. Network Construction and Topological Analysis

Using the STRING website, we calculated and constructed the PPI network interaction diagram [34]. A network of disease-compound-target is used to elucidate the pathophysiology of MG using Cytoscape software (version 3.7.2) [35]. A fundamental network topology attribute called degree is utilized to assess the properties of various interventions [36]. Degree in an undirected graph is the number of nearby nodes. In an interactive network, betweenness is the quantity of shortest pathways between two nodes. We identified the essential nodes of the entire interaction network and comprehended the primary mechanism underlying its interaction effect by studying the degree and intermediate number [35]. Then the Gene Ontology Biological Process (GOBP) analysis method was applied to further investigate the biological characteristics of target genes.

### 2.6. Molecular Docking

The 3D structure of the ligand was imported from Protein Data Bank (PDB, https://www.rcsb.org/) [37], the ligands were then prepared, and the water was drained. Hydrogen and small molecule ligands were used to construct binding sites (active pockets), and molecular docking was conducted with active components.

### 2.7. In Vitro Experiments to Validate Compounds

We performed in vitro experiments with rat astrocytes (AS). AS was acquired from the ATCC cell bank (American Type Culture Collection, ATCC). AS is cultured in DMEM medium (containing 5% penicillin-streptomycin and 5% serum; DEME: Gibco; serum: tetrasodium serum; penicillin-streptomycin: Solarbio). Cells are stored in a clean cell culture incubator containing 5% CO_2_ at 37°C. AS were activated with 1 ug/ml of lipopolysaccharide (LPS) to form an inflammatory model and treated with 10 uM kaempferol. The effect of kaempferol on specific target genes, including STAT3, MAPK1, HSP90AA1, PIK3R1, PIK3CA, and RXRA, was observed.

### 2.8. Quantitative PCR

RNA from astrocytes in 6-well plates was extracted using the Axygen RNA kit (Amresco, China) according to the manufacturer's instructions. The concentration and purity of the RNA, extracted cellular RNA, was measured using a spectrophotometer. Total RNA was reverse transcribed to cDNA using the TARAKA cDNA synthesis kit (Takara Bio) according to the manufacturer's instructions.

We have designed gene-specific primer sequences based on the target genes (STAT3, MAPK1, HSP90AA1, PIK3R1, PIK3CA, and RXRA) on the NCBI website (https://www.ncbi.nlm.nih.gov/) and the primer sequences are arranged in Table 1. The cells were extracted from intracellular RNA by adding cell lysate and other relevant operations after the drug concentration was applied. After the reverse transcription of RNA to cDNA was completed, specific primers were added to the 96-well plates to co-act with the intracellular cDNA, which was analyzed and observed by quantitative PCR to see if the drug was regulated through these targets of SCI and drug coactivity.

## 3. Results

### 3.1. Potential Active Compounds Identification

We extract 11 MG of active compound candidates from the TCMSP database (OB ≥ 30%, DL ≥ 0.18) [30]. However, no eligible components can be found in the BATMAN-TCM database [26]. The details of database searches and data extraction are shown in Table 2. We explored the research on the role of each major component. Among them, M01 beta-sitosterol has many biological functions, such as antianxiety and sedation, analgesia, immunomodulation, antibacterial, anticancer, anti-inflammatory, lipid-lowering, liver protection, protection against NAFLD, wound healing, antioxidant, and antidiabetic activities [38]. M02 kaempferol (3,5,7-trihydroxy-2 -(4-hydroxyphenyl) -4H-chromen4-one) is a polyphenol that is abundant in fruits and vegetables. Kaempferol has been reported to exert anti-inflammatory effects differently [39]. It can reduce oxidative stress and inflammatory response and promotes recovery of motor function in rats with SCI [23]. M03 mandenol is an unsaturated fatty acid with antibacterial and anti-inflammatory properties, which is used in many cosmetics [4]. M04 Supraene is one of the metabolic intermediates in the sterol biosynthesis pathway and has the effect of anti-oxygen free radical [40]. M06 perlolyrine is a beta-carboline alkaloid from *Codonopsis pilosula* [41]. M10 (S)-2-methylbutyl-4-(4-decyloxybenzylideneamino) was used for liquid crystal stationary phase (LCSP) analysis [42].

### 3.2. MG-Related Targets and SCI-Related Targets

To directly retrieve the name of the active ingredient's target gene, we entered the SMILE number of the active components (UniProt ID) into the SwissTargetPrediction platform [30] and deleted the duplicated value. For the active ingredients whose SMILE number cannot be obtained directly from the website, we use Open Babel software to calculate [43]. Finally, analyzing the above data, we received 11 MG-related active compounds and 346 MG-related targets.

The GeneCards database (https://www.genecards.org/) was performed to predict the potential targets of compounds, and 5716 potential targets were obtained [31]. Then, we used the same strategy to examine the Comparative Toxicogenomics Database (CTD, https://ctdbase.org/) for 3234 possible targets [32]. In addition, we obtained 15 potential targets of SCI from the DrugBank database (https://go.drugbank.com/) [33]. After converting the potential targets from the three databases into UniProt numbers and merging them, 7214 potential targets remained after the duplicate values were removed.

### 3.3. Therapeutic Targets for MG-Anti-SCI

**Figure 2:**
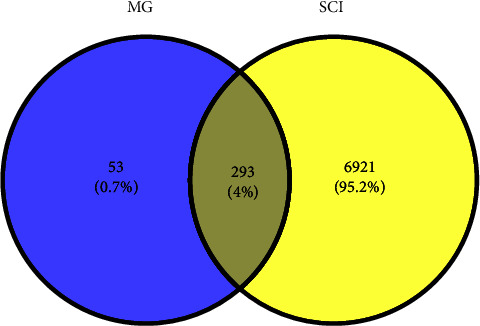
Venn diagram for MG-related targets and SCI-related targets.

We discovered the therapeutic targets at the confluence of MG and SCI, which had potential therapeutic benefits for treating SCI with MG. Venny software was used to intersect the MG and SCI targets, as illustrated in Figure 2, and 293 prospective targets were found [44].

**Figure 3:**
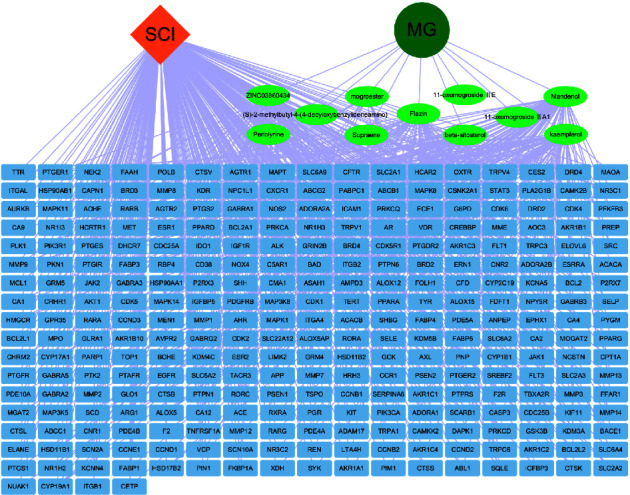
MG-active compounds-targetgenes-SCI network.

The interaction network is then constructed using Cytoscape software to reflect the connection between active compounds from MG and SCI targets (Figure 3). In Figure 3, the interaction relationship is shown by the line connecting the nodes.

### 3.4. Hub Genes of MG-Anti-SCI and Construction of PPI Network

**Figure 4:**
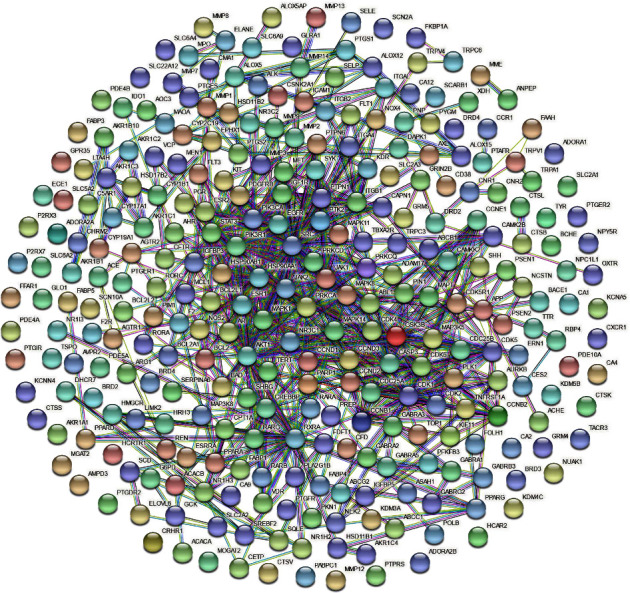
PPI network constructed with STRING.

We retrieved 293 therapeutic targets of MG for SCI from the STRING database and constructed a PPI network (Figure 4) consisting of 270 and 584 nodes, and the average node degree is 5.04. The TSV format file acquired from the STRING website is then imported into the Cytoscape program for additional analysis and visualization. The key genes for MG anti-SCI are determined from the preceding PPI network using the CytoNCA App plugin of the Cytoscape software. The sorting result of the top 10 hub genes (SRC, STAT3, MAPK1, HSP90AA1, PIK3R1, PIK3CA, RXRA, AKT1, CREBBP, and JAK2) is completely according to the algorithm of degree (Table 3).

### 3.5. GO and KEGG Pathway Enrichment Analysis

**Figure 5:**
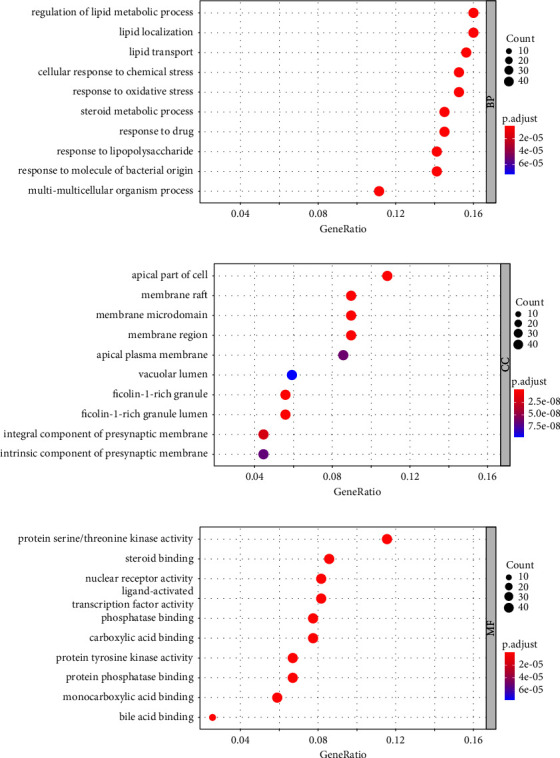
The bar chart of top 10 GO (BP, CC, and MF) enriched items. (a) Biological process (BP), (b) cellular component (CC), and (c) molecular function (MF).

GO and KEGG pathway enrichment analysis plays a significant role in network pharmacology research. Through the DOSE, enrichplot, path view, KEGGgraph, Rgraphviz, ClusterProfiler, org.Hs.eg.db, and ggplot2 packages in the R Studio software, we operated GO enrichment analysis on the 293 obtained targets of MG-anti-SCI. As a result, we received a total of 2781 GO items, including 2426 BP, 98 CC, and 255 MF. The top ten enrichment results of BP, CC, and MF are displayed using a bar chart or a bubble chart (Figure 5).

**Figure 6:**
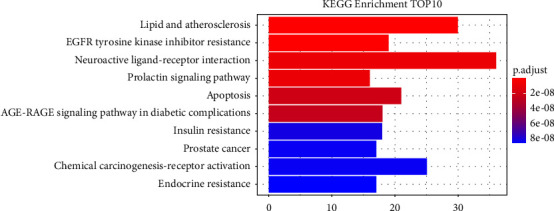
The bar chart of top 10 KEGG enriched pathways.

In the same way, through the ClusterProfiler, enrichplot, pathview, KEGGgraph, org.Hs.eg.db, ggplot2, and Rgraphviz packages in the R Studio software, using KEGG pathway enrichment analysis on 293 therapeutic targets, we identified 148 KEGG pathways. The top ten KEGG enrichment pathways are displayed in descending order, including lipid and atherosclerosis (hsa05417), EGFR tyrosine kinase inhibitor resistance (hsa01521), neuroactive ligand-receptor interaction (hsa04080), prolactin signaling pathway (hsa04917), apoptosis (hsa04210), AGE-RAGE signaling pathway in diabetic complications (hsa04933), insulin resistance (hsa04931), prostate cancer (hsa05215), chemical carcinogenesis-receptor activation (hsa05207), and endocrine resistance (hsa01522). The visualization of the above KEGG result is shown in Figure 6.

### 3.6. Molecular Docking between Hub Genes and Key Compounds

We performed molecular docking between the top 10 hinge genes and kaempferol (SRC, STAT3, MAPK1, HSP90AA1, PIK3R1, PIK3CA, RXRA, AKT1, CREBBP, and JAK2). The result of the molecular docking score, which ranged from 82.5427 to 116.96, is shown in Table 3.

**Figure 7:**
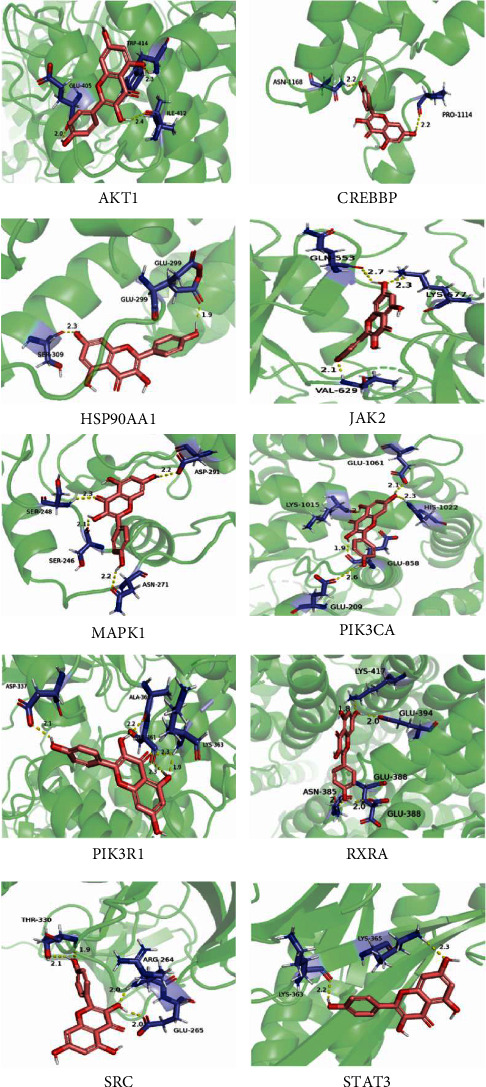
Molecular docking maps of 10 hub target genes to kaempferol.

The results show that kaempferol has good docking results with AKT1, RXRA, HSP90AA1, CREBBP, and JAK2. For instance, the scoring function LibDockScore of LibDock molecular docking is based on the ligand and receptor's affinity, relative energy, and docking mode. It is commonly assumed that a LibDockScore greater than 100 implies a stronger binding, and the higher the score, the more stable the shape of the complex formed via docking. The greater the affinity, the higher the ligand's binding strength to the protein (Table 4, Figure 7).

Based on the analysis of kaempferol and hub target data through molecular docking software, the diagram in Figure 7 is obtained.

### 3.7. The Results of Quantitative PCR

**Figure 8:**
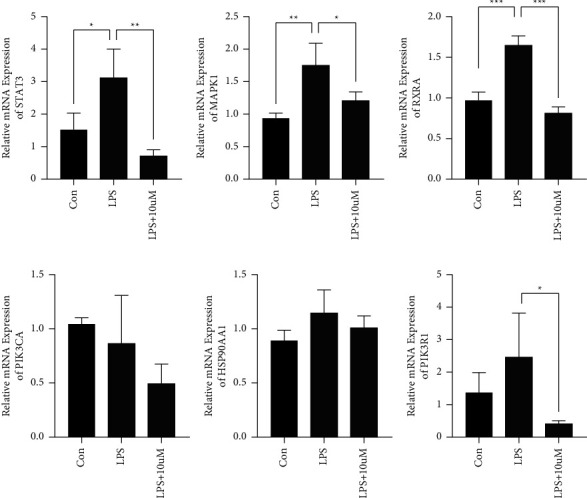
The relative mRNA expression of significantly different genes. (a) The relative mRNA expression of STAT3. (b) The relative mRNA expression of MAPK1. (c) The relative mRNA expression of RXRA. (d) The relative mRNA expression of PIK3CA. (e) The relative mRNA expression of HSP90AA1. (f) The relative mRNA expression of PIK3R1 (^*∗*^*P* < 0.05, ^*∗∗*^*P* < 0.01, and ^*∗∗∗*^*P* < 0.001 compared to LPS group).

The expression of hub gene targets (HSP90AA1, PIK3R1, PIK3CA, STAT3, RXRA, and MAPK1) was detected in the inner AS cell by real-time PCR (Figure 8). The divergence in the expression of relative mRNA (HSP90AA1, PIK3R1, and PIK3CA) between groups (LPS and LPS + 10 uM kaempferol) was not statistically significant by means of quantitative PCR. However, the expression of relative mRNA (STAT3, RXRA, and MAPK1) in LPS + 10 uM kaempferol group significantly falls down than that of LPS, and the difference was statistically significant.

## 4. Discussion

According to the report, the random aggregated yearly incidence of traumatic SCI in the Middle East and North Africa (MENA) Region was 23.24 cases per million individuals [45]. Individuals, families, and society bear huge costs due to these SCI patients. At the moment, significant progress has been made in the mechanism and clinical care plan of post-SCI, which includes decompression surgery, drugs, and rehabilitation. The prognosis for SCI patients has improved as a result of these actions. However, there is not enough randomized clinical research to demonstrate how well the recently identified repair approach improves SCI recovery [46]. Researchers are looking into pharmaceuticals, surgeries, medical bioengineering, and rehabilitation procedures to treat patients effectively. As is well known, TCM has been used in Asian countries to treat various diseases since ancient times [47]. MG is a traditional medicine used by Chinese people to treat respiratory tract infections. According to research, MG has anti-inflammatory properties and can lower blood lipids and scavenge oxygen free radicals [48, 49]. Kaempferol is one of the important active components of MG, which has been proven to be able to treat SCI in animal experiments [23]. However, considering its potential therapeutic value for SCI and its wide range of applications in daily life, the precise effect and specific mechanism of MG in treating SCI in humans are unclear. Therefore, we used the technology of network pharmacology to deeply study the mechanism of MG working on SCI and lay the groundwork for further research.

Initially, compounds and targets derived from MG were obtained by investigating TCMSP and BATMAN-TCM. OB ≥ 30 and DL ≥ 0.18 were the ADME principles. Then, relevant target proteins were acquired from the SwissTargetPrediction databases (probability > 0), and their UniProt IDs were validated on the UniProt platform.

The operational process of the TCMSP and BATMAN-TCM is separated into five steps: (1) The compounds of MG are directly obtained (TCMSP: 182, BATMAN-TCM: 0). (2) Screening out related compounds according to ADME standards (TCMSP: 11, BATMAN-TCM: 0). (3) The SwissTargetPrediction database is used to retrieve the target of the active component. The likelihood that is higher than 0 is the selected standard. (4) The final target is obtained by deleting duplicates (total number of targets: 346). (5) Checking for and recording the UniProt ID number of the obtained compound's target protein. Finally, 346 MG-related targets and 11 MG-related active molecules were identified.

In the second step, 7214 SCI-related targets were acquired by exploring 3 disease databases, including CTD (number: 3234), GeneCards (number: 5716), and DrugBank (number: 15). The website's online tool then constructs a Venn diagram of MG and SCI-related targets and extracts 293 targets from MG that may be beneficial in treating SCI.

Finally, the top ten hub genes (SRC, STAT3, MAPK1, HSP90AA1, PIK3R1, PIK3CA, RXRA, AKT1, CREBBP, and JAK2) were determined to have a higher therapeutic value for MG against SCI and will be examined subsequently.

AKT1 phosphorylates and activates the downstream pathway during SCI. The PI3K/Akt signaling pathway can help patients recover from acute SCI [50]. Our findings imply that AKT1 plays an important role in SCI healing. These findings are consistent with the results of earlier investigations [51].

To lower oxidative stress and inflammatory response, kaempferol can downregulate ROS-dependent MAPKs, NF-*κ*B, and the pyroptosis signaling pathway, which shows that the active compound candidate kaempferol offers potential for the treatment of SCI [23]. The molecular docking analysis results revealed.

Molecular docking methods are generally considered useful techniques for screening drug candidates [52]. Molecular docking techniques can predict affinity and binding feasibility between candidate compounds and target proteins. The score of molecular docking is determined by the interplay of Van der Waals forces, Coulomb interactions, and hydrogen bonds. It is one of the primary indicators used to evaluate the binding ability of target proteins to potential ligands. According to molecular docking experiments, MG kaempferol compounds have a high affinity for various SCI-related target proteins.

Through go enrichment analysis, we obtained the top 10 target genes of 293 targets of MG-anti-SCI, related biological processes are as follows: cellular response to chemical stress (GO-BP:0062197), lipid transport (GO-BP:0006869), steroid metabolic process (GO-BP:0008202), regulation of lipid metabolic process (GO-BP:0019216), lipid localization (GO-BP:0010876), response to lipopolysaccharide (GO-BP:0032496), response to molecule of bacterial origin (GO-BP:0002237), response to drug (GO-BP:0042493), response to oxidative stress (GO-BP:0006979), multi-multicellular organism process (GO-BP:0044706), nuclear receptor activity (GO-MF:0004879), ligand-activated transcription factor activity (GO-MF:0098531), steroid binding (GO-MF:0005496), monocarboxylic acid binding (GO-MF:0033293), protein serine/threonine kinase activity (GO-MF:0004674), phosphatase binding (GO-MF:0019902), protein tyrosine kinase activity (GO-MF:0004713), carboxylic acid binding (GO-MF:0031406), protein phosphatase binding (GO-MF: 0019903), bile acid binding (GO-MF:0032052), apical part of cell (GO-CC:0045177), membrane raft (GO-CC:0045121), membrane microdomain (GO-CC:0098857), membrane region (GO-CC:0098589), ficolin-1-rich granule (GO-CC:0101002), ficolin-1-rich granule lumen (GO-CC:1904813), integral component of presynaptic membrane (GO-CC:0099056), apical plasma membrane (GO-CC:0016324), intrinsic component of presynaptic membrane (GO-CC:0098889), and vacuolar lumen (GO-CC:0005775).

The top 10 KEGG pathway enrichment analysis results are provided together with 293 MG targets against SCI. The enriched pathways were categorized, and 10 highly associated pathways were selected as follows: lipid and atherosclerosis (hsa05417), EGFR tyrosine kinase inhibitor resistance (hsa01521), neuroactive ligand-receptor interaction(hsa04080), prolactin signaling pathway (hsa04917), apoptosis (hsa04210), AGE-RAGE signaling pathway in diabetic complications (hsa04933), insulin resistance (hsa04931), prostate cancer (hsa05215), chemical carcinogenesis-receptor activation (hsa05207), and endocrine resistance (hsa01522).

Based on our preliminary findings from the above network pharmacology study, we believe that the primary active components of MG may play a role in the treatment of SCI via the pathways mentioned above and hub genes. Taken together, we anticipate that an increasing number of researchers will commit themselves to clarify the mechanism of TCM's action through network pharmacology, thereby increasing global awareness of this Chinese treasure. In the future, based on the above research results, we will explore the effects of MAPK1, RXRA, and STAT3 on SCI through more *in vivo* and *in vitro* experiments.

## Figures and Tables

**Table 1:** Primer sequences for the target genes.

Gene	Preprimers	Postprimers
STAT3	CTAACCGGATCGCTGAGGTA	ATCTGCTGCTTCTCCGTCAC
MAPK1	AGTGCCTTCTGACTTTCCTGG	ATGCATGGCAAAGGACACCA
HSP90AA1	TTCATCAGAGGGGTGGTGGA	CACCAAGGTCTTGCCCTCAA
PIK3R1	GTCGTAGCTGGATCGAAGGA	CAATATCTTCTGGCCGGGCT
PIK3CA	CTGCAGTTCAACAGCCACAC	CCAGCTGCCATCTCAGTTCA
RXRA	CACCAAACATTTCCTGCCGC	TAGTGTTTGCCTGAGGAGCG

**Table 2:** Pharmacokinetic parameters of candidate active compounds in *Momordica grosvenori*.

Drug ID	Molecule name	SMILES	OB (%)	DL (%)
M01	Beta-sitosterol	CCC(CCC(C)C1CCC2C1(CCC3C2CC=C4C3(CCC(C4)O)C)C)C(C)C	36.91	0.75
M02	Kaempferol	C1=CC(=CC=C1C2=C(C(=O)C3=C(C=C(C=C3O2)O)O)O)O	41.88	0.24
M03	Mandenol	CCCCCC=CCC=CCCCCCCCC(=O)OCC	42	0.19
M04	Supraene	CC(=CCCC(=CCCC(=CCCC=C(C)CCC=C(C)CCC=C(C)C)C)C)C	33.55	0.42
M05	ZINC03860434	No result	43.59	0.35
M06	Perlolyrine	C1=CC=C2C(=C1)C3=C(N2)C(=NC=C3)C4=CC=C(O4)CO	65.95	0.27
M07	Flazin	C1=CC=C2C(=C1)C3=CC(=NC(=C3N2)C4=CC=C(O4)CO)C(=O)O	94.28	0.39
M08	11-Oxomogroside IIA1	CC(CCC(C(C) (C)O)OC1C(C(C(C(O1)COC2C(C(C(C(O2)CO)O)O)O)O)O)O)C3CCC4(C3(CC(=O)C5(C4CC=C6C5CCC(C6(C)C)O)C)C)C	37.63	0.22
M09	11-Oxomogroside IIE	No result	32.77	0.21
M10	(S)-2-Methylbutyl-4-(4-decyloxybenzylideneamino)	CCCCCCCCCCOC1=CC=C(C=C1)C=NC2=CC=C(C=C2)C=CC(=O)OCC(C)CC	45.01	0.71
M11	Mogroester	No result	41.69	0.31

Oral bioavailability (OB) > 30 and drug-like properties (DL) > 0.18.

**Table 3:** Top 10 nodes in the network ranked by degree.

Rank	Name	Degree
1	SRC	102
2	STAT3	74
3	MAPK1	66
4	HSP90AA1	64
5	PIK3R1	56
6	PIK3CA	56
7	RXRA	56
8	AKT1	54
9	CREBBP	52
10	JAK2	48

**Table 4:** LibDockScore of kaempferol with the top 10 key genes.

Ligand	Protein	LibDockScore
Kaempferol	AKT1	116.96
Kaempferol	RXRA	115.034
Kaempferol	HSP90AA1	107.893
Kaempferol	CREBBP	106.266
Kaempferol	JAK2	103.258
Kaempferol	PIK3CA	99.4724
Kaempferol	SRC	98.1891
Kaempferol	PIK3R1	96.2682
Kaempferol	MAPK1	94.0172
Kaempferol	STAT3	82.5427

## Data Availability

The data used to support the findings of this study are included within the article.
